# Proteome-Wide Detection and Annotation of Receptor Tyrosine Kinases (RTKs): RTK-PRED and the TyReK Database

**DOI:** 10.3390/biom13020270

**Published:** 2023-02-01

**Authors:** Georgios Filis, Fotis A. Baltoumas, Georgios Spanogiannis, Zoi I. Litou, Vassiliki A. Iconomidou

**Affiliations:** 1 Section of Cell Biology and Biophysics, Department of Biology, School of Sciences, National & Kapodistrian University of Athens, 15701 Athens, Greece; 2 Institute for Fundamental Biomedical Research, Biomedical Science Research Center “Alexander Fleming”, Vari, 16672 Athens, Greece

**Keywords:** databases, algorithms, classification, membrane proteins, machine learning, receptor tyrosine kinases (RTKs)

## Abstract

Receptor tyrosine kinases (RTKs) form a highly important group of protein receptors of the eukaryotic cell membrane. They control many vital cellular functions and are involved in the regulation of complex signaling networks. Mutations in RTKs have been associated with different types of cancers and other diseases. Although they are very important for proper cell function, they have been experimentally studied in a limited range of eukaryotic species. Currently, there is no available database for RTKs providing information about their function, expression, and interactions. Therefore, the identification of RTKs in multiple organisms, the documentation of their characteristics, and the collection of related information would be very useful. In this paper, we present a novel RTK detection pipeline (RTK-PRED) and the Receptor Tyrosine Kinases Database (TyReK-DB). RTK-PRED combines profile HMMs with transmembrane topology prediction to identify and classify potential RTKs. Proteins of all eukaryotic reference proteomes of the UniProt database were used as input in RTK-PRED leading to a filtered dataset of 20,478 RTKs. Based on the information collected for these RTKs from multiple databases, the relational TyReK database was created.

## 1. Introduction

Kinases are enzymes that catalyze the transfer of a phosphate group from a high-energy phosphate donor molecule (e.g., ATP) to a receptor substrate. By categorizing kinases according to the nature of their substrates, they can be divided into protein kinases, which phosphorylate peptides or proteins, and to non-protein kinases, which phosphorylate nucleotides, lipids, sugars, and other small molecules [1].

Protein kinases can be further divided into groups, based on the type of amino acid residue they phosphorylate. These groups primarily include Serine/Threonine (Ser/Thr) kinases, which phosphorylate Ser and Thr residues, Tyrosine (Tyr) kinases, which phosphorylate Tyr residues, and dual-specificity kinases, which can act as both Ser/Thr and Tyr kinases. The aforementioned groups share significant sequence and structural similarities in their catalytic domain and correspond to approximately 2–3% of the protein-encoding genes in humans and other model organisms [2,3], making them one of the largest protein groups in the human proteome. Other protein kinase groups also exist (e.g., histidine, aspartate, arginine, lysine kinases) which share limited similarities with Ser/Thr, Tyr, and dual-specificity kinases and adopt different enzyme mechanisms.

The group of tyrosine kinases is further divided into “receptor tyrosine kinases” (RTKs) and “non-receptor tyrosine kinases” (“nonRTKs”) or else “cytoplasmic tyrosine kinases”. There are 58 well-studied and documented RTKs in the human proteome, based on which a categorization of 20 different subfamilies (Table 1) has emerged [4,5,6,7]. The latter categorization was followed in the current scientific study. In addition, almost all known RTKs share the following specific traits:

- Signal peptide: The majority of RTKs are single-pass type I membrane proteins featuring an extracellular N-terminus, a single transmembrane (TM) segment and a cytoplasmic C-terminus. These proteins contain a signal peptide at their N-terminal region through which they initially attach to the membrane of the endoplasmic reticulum (ER). After the N-terminal region of the protein is embedded into the ER lumen, a signal peptidase cleaves the signal peptide from the mature protein [8,9,10].
- Extracellular domains: The N-terminal region of RTKs is in the extracellular matrix and usually contains several extracellular domains which interact with a variety of signaling molecules (ligands). Generally, the interactions between the extracellular domains of the RTK with ligands lead to its structural rearrangement and activation [7].
- Transmembrane (TM) domain: RTKs have only one transmembrane domain which is an α-helix, thus RTKs are single spanning proteins. It stabilizes the receptors to the membrane, and it has a significant role in the dimerization of the RTK monomers. The structure and orientation of the TM domain determine the structural position of the juxtamembrane. It affects the tyrosine kinase domain towards being activated or inactivated [4,11].
- Juxtamembrane domain: This domain is about 40 to 80 amino acids long [12] in ErbB and generally consists of several basic and positively charged amino acid residues. It interacts with the hydrophobic lipids of the membrane, especially with the PIP_2_ lipids, increasing the concentration of the latter ones into specific regions of the membrane. The PIP_2_ lipids, in certain concentrations, can act as signaling factors affecting a variety of functions in the cell [13]. Furthermore, the juxtamembrane region can act as an autoinhibitory element upon the tyrosine kinase domain [14].
- Tyrosine kinase domain (TKD): It is comprised of approximately 250 amino acids [12] and it is a highly conserved domain between the eukaryotic species. It also shares certain conserved features with Ser/Thr kinase domains. The branch of Tyr kinases, from an evolutionary perspective, is considered to have arisen from Ser/Thr kinases. The similarities between these two groups of kinases further complicate the process of prediction algorithms to distinguish between the two [15,16].
- C-terminal region: It can play a role in the activation and deactivation of the TKDs in certain types of RTKs, such as in the case of EGFR. A similar case is that of the C-terminal region of Tie2 that contains certain tyrosine autophosphorylation sites which can block the substrate from accessing the active site. Autophosphorylation in the C-terminal region of Tie2 could lead to the disruption of these autoinhibitory forces [7]. Another example showing the significance of tyrosine autophosphorylation sites in the C-terminal region of RTKs is the case of FGFR1. In the C-terminal region of FGFR1, a tyrosine autophosphorylation site is used as a high-affinity binding site for the SH2 domain of phospholipase Cγ (PLCγ). Based on the interaction of PLCγ with this autophosphorylation site, PLCγ is phosphorylated and activated by FGFR1 [15]. Furthermore, the C-terminal region, in the cases of MET and RON, comes into contact with the active site of the TKD leading to the inhibition of substrate access and stabilization of the inactive conformation of the TKD [17].

**Table 1:** The 20 subfamilies of the 58 human RTKs. The table contains the name(s) of each subfamily, the members of each subfamily and the main extracellular domains found in the members of each subfamily.

No.	Subfamily	Members	Extracellular Domains
1	EGFR	EGFR, ERBB2, ERBB3, ERBB4	L-domain ^1^, Furin-like Cysteine-rich ^2^
2	INSR	INSR, IGF1R, INSRR	L-domain, Furin-like Cysteine-rich, Fibronectin type III ^3^
3	PDGFR	PDGFRα, PDGFRβ, CSF1R/FMS, KIT/SCFR, FLT3/FLK2	Immunoglobulin-like ^4^ (5 repeats)
4	VEGFR	VEGFR1/Flt1, VEGFR2/KDR, VEGFR3/Flt4	Immunoglobulin-like (7 repeats)
5	FGFR	FGFR1, FGFR2, FGFR3, FGFR4	Immunoglobulin-like (3 repeats), Acid box
6	PTK7/CCK4	PTK7/CCK4	Immunoglobulin-like (7 repeats)
7	TRK	TRKA, TRKB, TRKC	Immunoglobulin-like, Leucine-rich ^5^
8	ROR	ROR1, ROR2	Immunoglobulin-like, Fz ^6^, Kringle ^7^
9	MUSK	MUSK	Immunoglobulin-like (3 repeats), Fz
10	HGFR/c-MET	MET, RON	Sema ^8^, IPT/TIG ^9^ (4 repeats), Psi
11	AXL/TAM	AXL, MER, TYRO3	Immunoglobulin-like (2-repeats), Fibronectin type III (2 repeats)
12	TIE	TIE1, TIE2	Immunoglobulin-like (3 repeats), Fibronectin type III (3 repeats), EGF (3 repeats)
13	EPH	EPHA1–8, EPHA10, EPHB1–4, EPHB6	Ephrin binding ^10^, Fibronectin type III (2 repeats)
14	RET	RET	Cadherin ^11^ (4 repeats), Furin-like Cysterine-rich
15	RYK	RYK	WIF ^12^
16	DDR	DDR1, DDR2	Discoidin ^13^ (2 repeats)
17	ROS	ROS	YWTD propeller ^14^ (3 repeats), Fibronectin type III (8 repeats)
18	LMR	LMR1, LMR2, LMR3	-
19	ALK	ALK, LTK	Mam ^15^ (2 repeats), Ldla ^16^
20	STYK1	SuRTK106/STYK1	-

Domain name: Pfam ID(s). ^1^ L-domain: PF01030, ^2^ Furin-like Cysteine-rich: PF00757, ^3^ Fibronectin type III: PF00041, ^4^ Immunoglobulin-like: PF00047, PF13895, PF13927, ^5^ Leucine-rich: PF13855, ^6^ Fz: PF01534, ^7^ Kringle: PF00051, ^8^ Sema: PF01403, ^9^ IPT/TIG: PF01833, ^10^ Ephrin binding: PF01404, ^11^ Cadherin: PF00028, ^12^ WIF: PF02019, ^13^ Discoidin: PF00754, ^14^ YWTD propeller: PF00058, ^15^ Mam: PF00629, ^16^ Ldla: PF00057.

The activation process of the RTKs is based on the enhancement of the catalytic activity of their TKDs. In general, RTKs exist as monomers on the membrane, bind ligands through their extracellular domains, and proceed to oligomerize with other RTKs [5]. In other cases, RTKs (e.g., IGF1R, EGFR) may have already formed oligomers upon the surface of the membrane but their TKDs are still not fully activated. The binding of their extracellular domains to ligands leads to rearrangements of their TKDs that lift the inhibition forces acting upon them. Regions of the RTKs which are phosphorylated at specific tyrosine residues during their activation, are identified from specific domains of cytoplasmic proteins. The latter proteins are responsible for downstream signal transduction and activation of one or more signaling cascades which in turn regulate highly important functions of the cells.

The major role of RTKs in numerous cell functions explains their association with multiple diseases. Mutated RTKs and in general dysregulation of the RTK signaling pathways, have been correlated with autoimmune diseases, developmental syndromes, malignancies, and different types of cancers. Therefore, RTKs are of high pharmaceutical value and have been targets for numerous drugs designed for clinical scenarios regarding targeted therapies. Moreover, they have been the basis of research for new potential drugs [6,17,18,19,20,21,22].

To date, RTKs have been experimentally studied in some reference eukaryotic organisms and even in those cases, the structure and function of all members of their family have not been fully elucidated and comprehended. Therefore, there is a need to identify and analyze RTKs in a broader range of organisms as well as to document and organize the various information available about them in a database. Currently, neither a tool able to identify RTKs and classify them into their subfamilies nor a database with various types of information for RTKs is available.

Consequently, we created a new tool (RTK-PRED) able to determine whether a protein is an RTK and we implemented it in order to search for RTKs in all reference eukaryotic proteomes of the UniProtKB database. Then, we documented information for the identified RTKs from several databases and organized it in the new Receptor Tyrosine Kinases Database (TyReK-DB). Finally, we constructed a website to provide access to the data of the TyReK database.

## 2. Materials and Methods

### 2.1. The RTK-PRED Detection Algorithm

The RTK-PRED workflow involves three steps: (i) detection of the protein tyrosine kinase (PTK) domain, (ii) prediction of transmembrane topology, and (iii) detection of extracellular domains and classification of the receptor. The final output includes the classification of the input sequences as RTKs, non-receptor PTKs, or non-tyrosine kinases, the position of the PTK domain(s) in the case of identified PTKs, and, finally, functional classification and full description of the receptor’s topology for predicted RTKs.

In the first step, the input sequences are scanned using HMMER [23] against a newly created Hidden Markov Model profile (pHMM) for the PTK domain. If a sequence contains the domain, it is recognized as a PTK, otherwise, it is classified as a non-PTK. It should be noted that Pfam [24] currently contains a model for the tyrosine kinase domain (Pfam: PF07714); however, this model fails to discriminate between Tyr-kinases and their homologs, Ser/Thr-kinases and, in recent versions of Pfam, the two families have been merged into a single entry (Tyr-Ser/Thr kinases). To solve this issue and avoid the misclassification of non-PTKs as PTKs or RTKs, a new pHMM was constructed for the PTK domain, capable of discriminating between Tyr-kinases and Ser/Thr-kinases. This pHMM was created using HMM-ModE [25], a training protocol that uses both a positive and a negative training set, to facilitate the optimization of emission probabilities for the final pHMM. Briefly, the training protocol involved the following steps. The sequences of both training sets were initially grouped into clusters based on their sequence identity, using BLAST [26] to perform pairwise alignments and the Markov Clustering (MCL) algorithm [27] to cluster the results. The positive training set, as well as the produced clusters were used to construct preliminary profiles, which were then subjected to profile–profile alignments with MUSCLE [28]. The alignment results were used to identify discriminating residue positions in the profile of the positive training set and modify their emission probabilities, by calculating the relative entropy of each position and comparing it against a null model based on the negative training set. The calculations were performed using a heuristic model compatible with the Plan 7 architecture of HMMER. The resulting pHMM was finally subjected to a 10-fold cross-validation, to calculate the profile’s discriminatory thresholds (gathering, trusted and noise cut-offs), in accordance with Pfam standards [24,25]. 

In the second step, positive hits are initially checked for the number of detected PTK domains. Sequences containing only one copy of the PTK domain are submitted for transmembrane topology prediction. If the sequence contains an RTK-compliant topology (i.e., an extracellular N-terminus, one transmembrane α-helix and a cytoplasmic C-terminus containing a PTK domain), it is classified as an RTK. Sequences not having this topology, as well as sequences with multiple PTK domains, are classified as non-receptor PTKs. Transmembrane topology prediction is performed using Phobius [29], a fast, HMM-based method for the combined prediction of signal peptides and α-helical transmembrane domains.

In the final step, the detected RTKs are scanned for the detection of extracellular (EC) regions and are classified into subfamilies. EC regions are detected using HMMER and a library of Pfam [24] pHMMs corresponding to these domains (Table 1). RTK classification is primarily performed using the Pfam pHMMs; however, an exception is made for the PDGF, VEGF, FGF, CCK, TIE, and TAM subfamilies. These particular subfamilies all feature the same types of domains in repeats, with a different number of repeats for each subfamily [7] (Table 1). However, in several cases, the number of predicted repeats for the EC pHMMs was incorrect. To solve this issue, an additional set of pHMMs was constructed for these particular subfamilies, modeling the juxtamembrane (JM) sequence connecting the transmembrane segment with the PTK domain. These models are used alongside the EC pHMM library for the classification of these five subfamilies. The JM pHMMs were also trained using the HMM-ModE protocol in a way similar to the PTK pHMM. An additional exception is given for the XVIII and XX subfamilies, which have very small extracellular sequences without any domains [7]. Detection of RTKs from these subfamilies is performed by measuring the length of the extracellular N-terminus in RTKs without any EC domains. However, as RTK-PRED is unable to discriminate between these two subfamilies, they are grouped together into the same category (Subfamily XVIII or XX).

The positive and negative training sets for the PTK domain and JM regions were assembled by collecting the relevant sequence segments from well-annotated kinases, retrieved from UniProtKB/Swiss-Prot [30]. For the PTK domain pHMM, the positive training set was comprised of tyrosine kinase domain sequences, both from RTKs and from non-receptor tyrosine kinases, while the negative training set was comprised of the kinase domain sequences of other kinase types (Ser/Thr, His, non-protein kinases). The annotation of each sequence as a PTK or a non-PTK was based on their assignment in UniProtKB records, as well as the “Kinases” section of the IUPHAR/BPS Guide to Pharmacology [31]. Both sets had a maximum sequence identity cut-off of 40%, achieved by performing sequence filtering with CD-HIT [32]. For the JM region pHMMs, the juxtamembrane sequence regions of human and mouse RTKs were used to construct the training sets, with the sequences of each subfamily used as the positive set for that subfamily, whereas the rest of the sequences were used as the negative set.

The validation of RTK-PRED was conducted on three levels: (i) evaluation of the custom PTK pHMM’s ability to recognize the PTK domain, (ii) evaluation of the entire RTK-PRED pipeline for the identification of RTKs, and (iii) evaluation of the classification of RTKs. To perform these evaluations, a number of positive and negative test sets were compiled. For the evaluation of the PTK domain, the positive test set was comprised of 373 tyrosine kinases (200 RTKs and 173 non-receptor PTKs), chosen based on their experimentally determined annotation in UniProtKB and the IUPHAR/BPS Guide to Pharmacology. The negative set was composed of 6000 non-tyrosine kinase proteins, i.e., other kinase types (Ser/Thr, His, etc.) and other protein classes (ion channels, other receptors, various enzymes, etc.). For the evaluation of the entire RTK-PRED pipeline, the 173 non-RTK kinases were moved to the negative dataset, resulting in a positive set of 200 RTKs and a negative set of 6173 non-RTKs. Finally, the RTK-PRED positive set was also used to evaluate RTK classification, by assigning each receptor to its respective subfamily based on the classification system of the IUPHAR/BPS [31]. All test sets were created using well-annotated sequences collected from UniProtKB/Swiss-Prot and reduced to a maximum sequence identity of 40% through clustering with CD-HIT. 

RTK-PRED has been implemented as a web server, available through http://bioinformatics.biol.uoa.gr/RTK-PRED/ (accessed on 29 January 2023). A standalone version of the pipeline is also available through GitHub: https://github.com/fbaltoumas/RTK-PRED/ (accessed on 29 January 2023). All training and test sets are available for download through the web server’s *Downloads* page.

### 2.2. RTK Dataset Compilation

**Figure 1:**
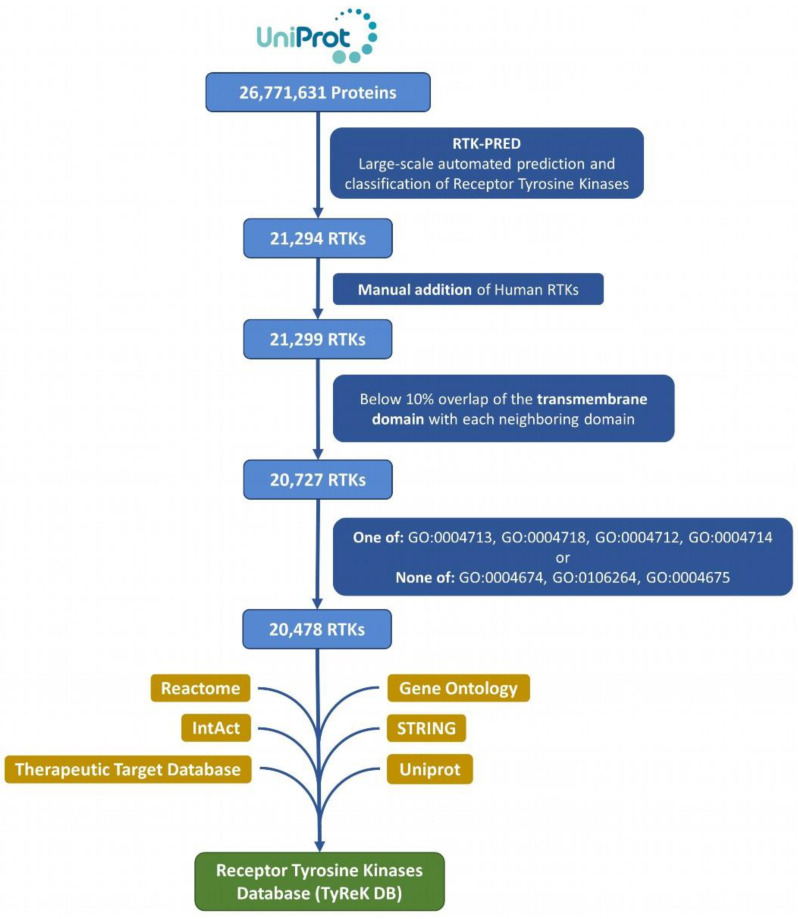
Flowchart for dataset collection and annotation in TyReK-DB.

All the reference eukaryotic proteomes (1511) of the UniProt database [30] (December 2020 release) were mapped to their proteins (26,771,631) in UniprotKB, which then were retrieved and analyzed (Figure 1). The protein sequences from the reference proteomes were analyzed with RTK-PRED, resulting in an initial set of 21,294 putative RTKs. This set was further enriched by manually inspecting the pipeline’s negative results for the existence of false negatives in the 58 human RTKs; this resulted in the addition of 5 human RTKs (UniProtKB ACs: P36888, Q01973, P54756, P29322, Q96Q04) and correction of misclassification of 2 human RTKs. The corrections included 1 RTK that belongs to the TIE subfamily and 1 RTK that belongs to the ALK subfamily (UniprotKB ACs: Q02763 and P29376 correspondingly). We do not consider as a misclassification the categorization of LMR or STYK1 RTKs in the “LMR or STYK1” group, but specifically for 3 human RTKs (UniprotKB ACs: Q6ZMQ8, Q8IWU2, Q6J9G0), whose subfamily is known, their classification changed specifically to LMR or STYK1. The set of RTKs was further filtered to remove any sequences with significant overlap between their TM segment with its neighboring EC domains, since residues forming the hydrophobic core of a large domain could potentially be mistaken as a TM segment and lead to false predictions. The overlap threshold was selected to be at least 10% of the TM domain’s length in order to exclude most of the proteins without any actual TM domains; any sequences violating this threshold were excluded. The domains of the 5 human RTKs that were added manually were predicted using the “*hmmscan*” utility of HMMER and included certain domains that potentially contain transmembrane regions, therefore the extension of the overlap in those cases was allowed at any level. The number of proteins excluded in this step of the analysis is 572. In addition, considering the significant similarity of the kinase domain among Tyr and Ser/Thr kinases, an additional filter was applied to exclude proteins without Tyr kinase activity and only with Ser or Thr or Ser/Thr kinase activity, based on their Gene Ontology terms [33,34], which were retrieved from a Gene Ontology Annotation (GOA) file [35]. From this filtering, 249 proteins were excluded and 20,478 RTKs remained, forming the final dataset of RTKs, which was used to construct the TyReK database. 

Furthermore, we submitted a query in UniProtKB in order to identify the proteins which most probably belong to the RTK family. The query aimed at searching for reviewed proteins which do not belong to the Tyrosine Kinase Like (TKLs) family of proteins and have, at the very least, the most basic traits of RTKs. These traits refer to the proteins being single-pass transmembrane and them being part of the family of protein tyrosine kinases. The query resulted in 330 proteins of which 254 were also identified in our final dataset of RTKs. Thus, another type of status emerged for the RTKs named “UniProt RTK status” (available in the search criteria of the website) that showed whether the RTKs identified by RTK-PRED also fulfilled the criteria of the query described above. Finally, based on the taxonomic lineage of each RTK and in order to reveal more information about the taxonomic groups of the organisms of the RTKs, several groups of RTKs were formed based on different taxonomic ranks.

The filtering based on the GO terms and all subsequent analysis of the RTKs are described in detail in the Supplementary Material, while the accession numbers of the excluded sequences are given in Supplementary File S2.

### 2.3. Data Annotation and Organization in TyReK-DB

Information for the final dataset of RTKs was collected from various databases. A detailed description is given in the Supplementary Material. RTK identifiers and metadata, including gene names, gene IDs [36], accession codes, taxonomy associations [37], and cross-references to major biological databases [38] were retrieved from UniProtKB. The sequences of the reference proteomes used as input in RTK-PRED were canonical sequences. For the proteins of the final dataset of RTKs, where available, the sequences of their isoforms were also retrieved from UniProtKB and added as a part of the information collected about them. The associated GO IDs were further annotated using the lists functionality of the YeastMine tool to obtain the names, namespaces, and definitions of the GO terms [39]. Metabolic and signaling pathway annotations were retrieved from Reactome [40]. Biomolecular interactions were retrieved from STRING [41] and IntAct [42,43]. RTK-drug associations were collected from the Therapeutic Target Database (TTD) [44]. The final data were organized in a new database named “Receptor Tyrosine Kinases Database (TyReK-DB)”. The backend of the database is supported by a MySQL relational database, with server-side operations being handled by PHP. The frontend is implemented with HTML, CSS and JavaScript and is available at http://bioinformatics.biol.uoa.gr/tyrek_db/ (accessed on 29 January 2023). The website contains multiple features and offers a range of utilities.

## 3. Results

### 3.1. RTK-PRED Evaluation

The validation of RTK-PRED was conducted on three levels: (i) evaluation of the custom PTK pHMM’s ability to recognize the PTK domain, (ii) evaluation of the entire RTK-PRED pipeline for the identification of RTKs, and (iii) evaluation of the classification of RTKs. For the first level, evaluation was performed using a positive dataset of PTKs and non-PTKs (Ser/Thr kinases, other kinase types, and other proteins) for the PTK pHMM. Results are given in Table 2. Results show that the custom pHMM performs adequately in the identification of the PTK domain (Sensitivity at 83.4%), while not erroneously detecting Ser/Thr-kinases or other kinase types in general (Specificity at 99.9%). As far as further discrimination of RTKs is concerned, the positive dataset was composed of well-annotated RTKs, while the negative dataset contained non-receptor PTKs and other protein types. As it can be seen (Table 2), the RTK-PRED pipeline performs well, with sensitivity at 93%, specificity at 99.8%, and an overall MCC value of 0.937. These results can be attributed not only to the performance of the custom PTK pHMM, but also to the use of the Phobius algorithm, which has been shown to perform well in predicting the correct topology for single-pass transmembrane proteins in general, including RTKs.

For the evaluation of RTK-PRED’s classification capabilities, the results of the positive test set were compared against the receptors’ classification in the IUPHAR/BPS Guide to Pharmacology [31]. Results are shown in Supplementary Table S1. As it can be seen, the pipeline’s classification system performs well, with 15/20 of the RTK subfamilies having very few false positives/negatives and showing sensitivity and specificity results larger than 90%. However, RTK-PRED performs poorly for the TIE and ALK subfamilies; this can be attributed to the small number of these proteins in the test set, as some TIE and ALK RTKs had been used in the training set of the PTK pHMM and/or the Pfam EC domain pHMMs. In the case of the LMR and STYK1 RTKs, which have short extracellular sequences with no domains, results are shown combined.

RTK-PRED has been implemented as a publicly available web server. Through the server’s home page, users can paste or upload one or multiple protein sequences in FASTA format, with a maximum of 100,000 sequences per run. Upon submission, each request is assigned a unique identifier, through which users can retrieve their job results from the “Retrieve Results” page. The “Help” page contains the pipeline’s user manual, including a description of the utilized methods and uses case examples. The “Downloads” page contains links for all the training and test datasets used in this study.

### 3.2. Summary Results of Identified RTKs

Initially, 26,771,631 proteins of all reference eukaryotic proteomes of the UniProt database were given as input to RTK-PRED and 21,294 RTKs were identified. After the preprocessing analysis, 20,478 RTKs remained and formed our final dataset.

**Figure 2:**
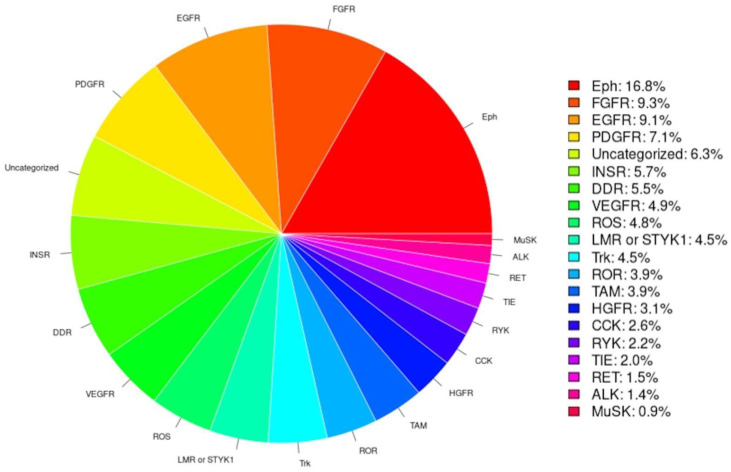
The pie chart is based on the classification of the RTKs by RTK-PRED, not including the manual addition nor the correction of the misclassifications of certain human RTKs.

RTK-PRED predicted correctly as RTKs 91% (53/58) of the 58 human RTKs and properly classified 96% (51/53) of the identified human RTKs. Based on the classifications of the identified RTKs by RTK-PRED (Figure 2), the subfamily with the highest percentage of RTKs is the Eph receptor subfamily, an expected outcome because this subfamily contains the most members compared to all other subfamilies. The class predicted to have the least RTKs was the MuSK subfamily. The TyReK database contains RTKs specifically of the LMR or STYK1 subfamilies which were added manually and also includes the corrections made regarding the categorization of two human RTKs.

**Figure 3:**
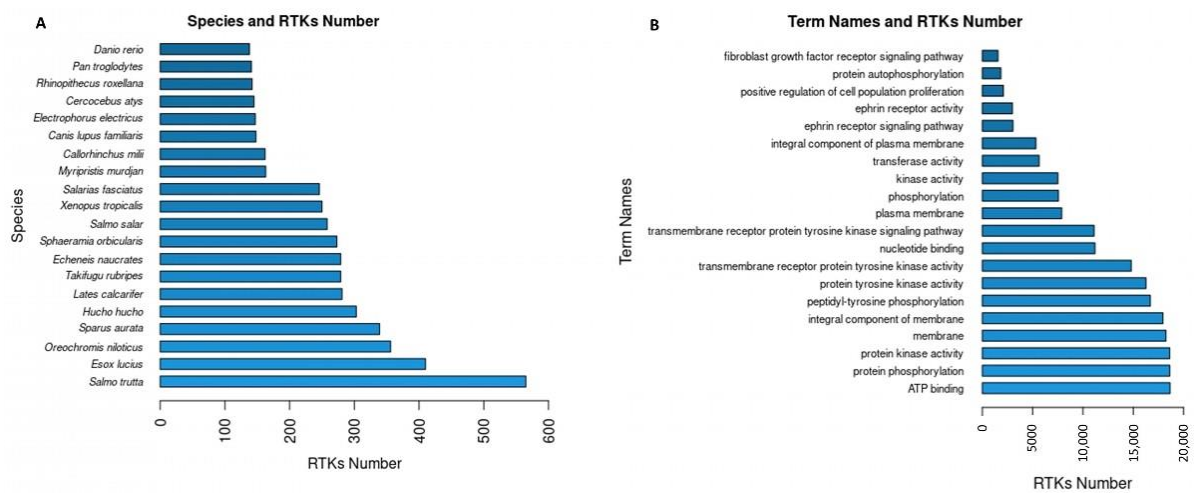
(**A**) The 20 most abundant in RTKs species based on the predictions of RTK-PRED and the NCBI taxonomy database; (**B**) the term names of the 20 GO IDs corresponded to the most RTKs.

The RTKs of the final dataset (20,478 RTKs) correspond to 445 different species. The 20 first species with the highest numbers in RTKs (Figure 3A) include species of interest since they are not identified as species generally used in studying RTKs (e.g., “*Salmo trutta*”, “*Esox lucius*”) neither experimentally nor computationally. Further analysis of the novel RTKs predicted by RTK-PRED in these species could reveal new information regarding their structural and functional aspects or their evolution among the different eukaryotic species. Overall, 1676 unique GO terms were found for 19,071 RTKs, 1668 of which corresponded to GO names, definitions, and domains by YeastMine. The GO names of the first 20 GO IDs (Figure 3B) that corresponded to the highest numbers of RTKs include information about the principal functions of RTKs and their cellular localization (e.g., “protein phosphorylation”, “membrane”). Similarly, 351 RTKs were identified in the data collected from the Reactome database and corresponded to 5493 unique pathways and reactions, 2773 RTKs were identified in the data collected from the STRING database and corresponded to 24,934 unique protein interactors, 153 RTKs were identified in the data collected from the IntAct database and corresponded to 9230 interactions, and 50 RTKs were identified in the information collected from the TTD and corresponded to 1041 unique drugs.

### 3.3. The TyReK Database

**Figure 4:**
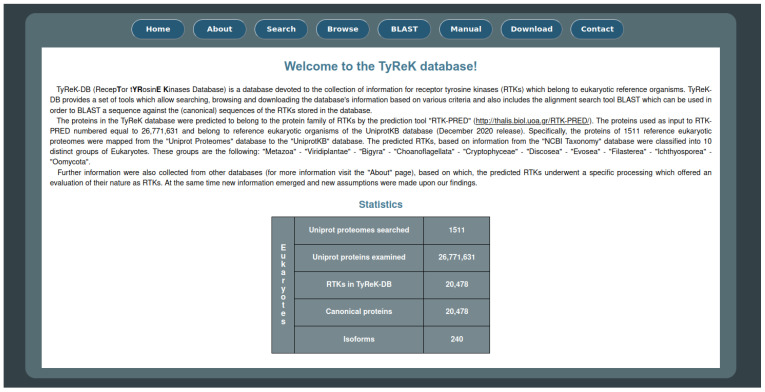
The main interface of the homepage of the TyReK-DB website.

The website for the TyReK database consists of eight main pages as shown on the homepage (Figure 4) and some additional pages regarding the results of the queries which can be submitted to the database. Some of the basic utilities that the website offers include the ability to search for RTKs based on various criteria, the ability to browse the RTKs based on different groupings of RTKs (e.g., subfamilies of RTKs, the species to which RTKs belong, domains which have been identified by RTK-PRED in the RTKs) and the BLASTp utility against the sequences of the RTKs in the TyReK database. The main and secondary pages of the TyReK-DB website are described in detail in the Supplementary Material.

## 4. Discussion

RTKs regulate multiple signaling pathways which in turn determine the response of the cell to extracellular signals (e.g., hormones) and affect fundamental cell functions. Thus, mutated RTKs or dysregulation of their function can lead to significantly altered cell phenotypes and have been correlated with numerous diseases. In our effort to automate the process of identifying RTKs alongside their subfamily and increase the current knowledge of them in a broad spectrum of species, we created RTK-PRED, a tool able to perform large-scale automated prediction and classification of RTKs. Furthermore, we implemented RTK-PRED in order to analyze proteins from all the eukaryotic reference proteomes of the UniProt database and after filtering the identified RTKs a dataset of 20,478 RTKs was formed. We collected information from various databases for these RTKs and finally we constructed the TyReK database and its website. Based on our analysis with RTK-PRED, RTKs were identified in 445 different species and the information collected for them includes aspects relative to their expression, subcellular location, function, involvement in signaling pathways and interactions with other proteins, molecules, and drugs. This information and the ability offered by the website of TyReK-DB to specifically select RTKs that fulfill certain criteria can be a crucial steppingstone in elucidating the evolutionary course of RTKs between the different eukaryotic species, the common functional and structural characteristics between RTKs of specific subfamilies, as well as the mechanisms underlying the regulatory nature of RTKs in signaling pathways. In addition, the association of RTKs with certain signaling cascades could also potentially be correlated with specific types of interactions with other proteins or molecules.

Although RTK-PRED and the TyReK database offer a rapid method of identifying and characterizing RTKs and a vast diversity of information collected for them with the ability to make complex searches based on different criteria for the RTKs setting the ground for future analysis of their nature and function, both come with certain limitations. RTK-PRED is capable of discriminating RTKs against non-receptor PTKs and other kinase types, as well as predicting RTK topology and classification. However, the latter is primarily based on evidence from humans and other model mammalian species and may not necessarily apply to other groups of organisms (e.g., insects, plants, or fungi). Furthermore, the algorithm was found to underperform in correctly predicting the classification of specific family members, such as ALK, ROS, LMR, and STYK1. This limitation can be attributed to the lack of enough sequence data for these particular subfamilies, which could enable the training of more specialized models. In addition, based on the implementation and use of RTK-PRED, the TyReK database contains information specifically for RTKs which have been identified in the proteomes of eukaryotic species. Moreover, searches in the TyReK database are based on a certain number of criteria while there is a large number of different kinds of information in the TyReK database. We are looking forward to increasing the functionality of the website, allowing more complex searches based on a greater number of criteria in correlation with all the kinds of information available for the RTKs in the TyReK database. Finally, the website currently does not offer a form in order to allow a user to directly submit their own findings related to RTKs. Such a form will be made available in future updates of the website.

## 5. Conclusions

The unknown mechanisms adopted by RTKs in order to perform a wide range of processes (e.g., activation, signaling) are yet to be fully elucidated. We believe that RTK-PRED and the TyReK database are tools that could significantly facilitate the process of discovering and classifying RTKs and organizing and utilizing the information available to them. Therefore, both tools can lead to a new scope of view of the novel and already known RTKs and give the chance to further understand their functions and involvement in signaling pathways.

In the future, we will consider adding a section in the TyReK database specifically focused on the ligands identified to be interacting with the extracellular domains of the RTKs. Moreover, a classification of these ligands could be performed based on the already-classified RTKs in their different subfamilies.

## Figures and Tables

**Table 2:** Validation of the custom PTK pHMM and RTK-PRED.

	PTK pHMM ^1^	RTK-PRED ^2^
**TP**	311/373	186/200
**TN**	5994/6000	6163/6173
**FP**	6/6000	10/6173
**FN**	62/373	14/200
**Sensitivity**	83.4%	93.0%
**Specificity**	99.9%	99.8%
**Accuracy**	98.9%	99.6%
**MCC**	0.899	0.937

^1^ Detection of the PTK domain (Positive Set: 373 PTKs, Negative Set: 6000 Non-PTKs (other kinases, other proteins)). ^2^ Prediction of RTKs (Positive set: 200 RTKs, Negative Set: 6173 Non-RTKs (Non-receptor PTKs, other kinases, other proteins)).

## Data Availability

RTK-PRED is available at http://bioinformatics.biol.uoa.gr/RTK-PRED/ (accessed on 29 January 2023). The source code is available at https://github.com/fbaltoumas/RTK-PRED/ (accessed on 29 January 2023). TyReK-DB is available through http://bioinformatics.biol.uoa.gr/tyrek_db/ (accessed on 29 January 2023).

## Supplementary Materials

The following supporting information can be downloaded at: https://www.mdpi.com/article/10.3390/biom13020270/s1, File S1: Supplementary Methods and Results [30,33,34,35,36,37,38,41,42,43,44]; File S2: Excluded and remaining UniprotKB ACs of RTKs for each step of the analysis of the TyReK-DB RTK dataset; Table S1: Validation of RTK-PRED’s classification. TP: True Positive, TN: True Negative, FP: False Positive, FN: False Negative, Sn: Sensitivity, Sp: Specificity, Acc: Accuracy, MCC: Matthews Correlation Coefficient.
