# Supplementary material for: Proteome-Wide Detection and Annotation of Receptor Tyrosine Kinases (RTKs): RTK-PRED and the TyReK Database
Source: Biomolecules. 2023 Feb 1;13(2):270. doi: 10.3390/biom13020270 (PMC9953206; doi:10.3390/biom13020270)
Supplement: Supplementary file 1 [file biomolecules-13-00270-s001.zip › Supplementary_File_S1.pdf]

Supplementary Material

Supplementary Material contents

1. Supplementary File S1 (this file): Supplementary Methods & Results
2. Supplementary File S2: Excluded and remaining UniprotKB ACs of RTKs for each step of the analysis of the TyReK-DB RTK dataset

Supplementary Methods

RTK filtering based on GO terms and further analysis

Certain Ser/Thr kinase domains have great similarities with Tyr kinase domains thus the process of classifying domains to the correct group between the two is not a simple task. For this reason, an additional filter was implemented to exclude proteins that potentially did not have Tyr kinase activity but only Ser or Thr or Ser/Thr kinase activity. This filter consisted of specific Gene Ontology (GO) terms [33, 34] based on which any proteins predicted to have only Ser or Thr or Ser/Thr kinase activity (GO:0004674, GO:0106264, GO:0004675) and no indication of Tyr kinase activity (GO:0004713, GO:0004718, GO:0004712, GO:0004714) were excluded from the dataset. The filter that was based on the GO terms consisted of specific Gene Ontology (GO) terms which referred to "protein serine/threonine kinase activity" (GO:0004674), "protein serine kinase activity (using GTP as donor)" (GO:0106264), "protein tyrosine kinase activity" (GO:0004713, GO:0004718), "protein serine/threonine/tyrosine kinase activity" (GO:0004712), "transmembrane receptor protein tyrosine kinase activity" (GO:0004714) and "transmembrane receptor protein serine/threonine kinase activity" (GO:0004675). In recent versions of GO the accession of GO:0004718 has been replaced by the accession of GO:0004713. The GO terms were initially collected from the Gene Ontology Annotation (GOA) [35] file for proteins in the UniProt KnowledgeBase (UniProtKB) [30] (gaf-version: 2.1, generated: 08/2020) based on the UniProtKB ACs of the RTKs. The GO terms for the 58 well studied human RTKs were later on collected based on the taxon-specific GOA file for the human organism provided by the GO Consortium (gaf-version: 2.2, generated: 03/2022) based on the UniProtKB ACs of the RTKs. From this filtering, 249 proteins were excluded and 20,478 RTKs remained, forming the final dataset of RTKs.

The protein products of a single gene for one species in the UniProtKB database include one "canonical" sequence. The canonical sequences of the proteins in UniprotKB, corresponding to the reference eukaryotic proteomes of Uniprot, were the ones to be analyzed by RTK-PRED and their corresponding isoforms are given as additional information for them (overall: 20,478 RTKs with 240 isoforms). We believe that it is highly probable that the isoforms of the proteins found to be RTKs by RTK-PRED are also RTKs themselves.

Furthermore, we submitted a query in UniProtKB in order to identify the proteins which most probably belong to the RTK family. The query aimed at searching for reviewed proteins which do not belong to the Tyrosine Kinase Like (TKLs) family of proteins and have, at the very least, the most basic traits of RTKs. These traits refer to the proteins being single-pass transmembrane and them being part of the family of protein tyrosine kinases. The query (based on the current advanced search system implemented by UniprotKB the corresponding query to the one made based on the previous advanced search system of UniprotKB would be: "(cc_scl_term:SL-9904) AND (family:"tyr protein kinase") AND (reviewed:true) NOT (family:tkl)") resulted in 330 proteins from which 254 were also identified in our final dataset of RTKs. Thus, another type of status emerged for the RTKs named "UniProt RTK status" that showed whether the RTKs identified by RTK-PRED also fulfilled the criteria of the query described above.

The RTKs were also grouped based on their rank in their taxonomic lineage, after the superkingdom of the organism they belong to, in order to reveal more information about the taxonomic groups of the organisms of the identified RTKs. By this means, 10 groups were formed based on different taxonomic ranks which are the following: Bigyra, Choanoflagellata, Cryptophyceae, Discosea, Evosea, Filasterea, Ichthyosporea, Metazoa, Oomycota and Viridiplantae.

Data collection and Receptor Tyrosine Kinases Database (TyReK-DB) construction

Based on the information available (30/12/2020) for the "lowest level pathway diagram/subset of the pathway" and the "database identifier to all reactions" from Reactome, the pathways and reactions in which RTKs, identified by RTK-PRED, were involved were collected.

The STRING database [41] contains known and predicted protein-protein interactions. Initially, the UniProtKB ACs of the RTKs were mapped to their STRING identifiers (if any). Then, the protein network data (scored links between proteins) specifically

for the organisms in which the RTKs had been found were collected. This data contained the combined score of each interaction in the network. The combined score is based on seven different types of evidence (conserved neighborhood, co-occurrence, fusion, co-expression, experiments, databases and text mining). Only the interactions with a combined score equal or above 0.7 were retained. The IntAct database [42,43] contains interactions which derive from literature curation or direct user submissions. The IntAct database was downloaded (latest version available at 01/01/2021) and was used to collect certain information for each RTK based on its UniProtKB AC. For every RTK, the name of the interactor, the interaction detection method(s), the 1st author(s) of the publication, the identifier(s) of the publication and the identifier(s) of the interaction were retained.

Based on the data provided by the Therapeutic Target Database (TTD) ("TTD targets information in raw format", Version 7.1.01, 2019.07.14) drugs were collected based on the UniProtKB IDs of the RTKs [44]. Three traits were retained for each protein, the TTD drug ID, the name of the drug and the highest clinical status.

From UniProtKB, specific information about different aspects of the RTKs was collected. These aspects, for each RTK, include its gene names, its gene ID from the database of genes of NCBI RefSeq genomes [36], its protein names, its proteome and chromosome identifiers, its state (reviewed or unreviewed), its taxonomic identifier and taxonomic lineage from the NCBI taxonomy database [37], its general functionality, the conditions or compounds which regulate its gene expression, the reactions it can catalyze, its regulatory mechanism, the developmentally-specific expression of its mRNA or protein, its subcellular location, the tissue-specific expression of its mRNA or protein, Bgee related data [38], its binding sites for any chemical group, its nucleotide phosphate-binding regions, its residues that take part in disulfide bonds, its residues that are modified at a post-translational stage, the chemical alternations which may occur to it, the diseases correlated with its deficiency, the effects caused by the disruption of its gene coding for the protein, the sites at which it has been experimentally altered by mutagenesis and cross references to other databases. The latter cross references lead to the following databases: EMBL, Bgee, BindingDB, BioMuta, BRENDA, CORUM, DIP, DisGeNET, DMDM, DrugBank, eggNOG, Ensembl, EuPathDB, ExpressionAtlas, GeneID, GeneReviews, Guide to PHARMACOLOGY, HPA, KEGG, MalaCards, MIM, MINT, PDB, PeptideAtlas, PhosphoSitePlus, RefSeq and TreeFam. In addition, the information collected for the RTKs for each of the aspects that were selected by UniprotKB, except of the ones originating from the NCBI taxonomy and the Bgee databases, includes its Evidence & Conclusion Ontologies (ECOs) and its corresponding sources. These sources consist of the literature (PubMed), UniProtKB and an automated process implemented by UniProtKB which performs automatic annotation of proteins.

Supplementary Results

Table S1. Validation of RTK-PRED's classification. TP: True Positive, TN: True Negative, FP: False Positive, FN: False Negative, Sn: Sensitivity, Sp: Specificity, Acc: Accuracy, MCC: Matthews Correlation Coefficient

Subfamily	TP	TN	FP	FN	Sn	Sp	Acc	MCC
**EGFR**	11	136	0	0	100	100	100	1.00
**INSR**	9	138	0	0	100	100	100	1.00
**PDGFR**	12	134	0	1	92.31	100	99.32	0.96
**VEGFR**	9	138	0	0	100	100	100	1.00
**FGFR**	10	137	0	0	100	100	100	1.00
**PTK7/CCK4**	2	145	0	0	100	100	100	1.00
**TRK**	9	138	0	0	100	100	100	1.00
**ROR**	2	143	0	2	50	100	98.64	0.70
**MUSK**	3	144	0	0	100	100	100	1.00
**HGFR/c-MET**	5	142	0	0	100	100	100	1.00
**AXL/TAM**	8	139	0	0	100	100	100	1.00
**TIE**	3	143	0	1	75	100	99.32	0.86
**EPH**	29	112	0	6	82.86	100	95.92	0.89
**RET**	2	145	0	0	100	100	100	1.00
**RYK**	2	145	0	0	100	100	100	1.00
**DDR**	5	142	0	0	100	100	100	1.00
**ROS**	3	143	1	0	100	99.30	99.32	0.86
**ALK**	2	143	0	2	50	100	98.64	0.70
**LMR or STYK1**	7	138	0	2	77.78	100	98.64	0.87

The TyReK-DB website

The main and secondary pages of the TyReK-DB website are described below:

- Home: It contains general information about the data available to the user through the website and statistical information about the proteins analyzed by RTK-PRED and the RTKs of the TyReK database.
- About: It provides a short description about the collection and filtering of the RTKs and about the accession numbers and their interpretation in the TyReK database.
- Search: The user can search for RTKs in the TyReK database through various criteria. These criteria include the accession numbers of the TyReK database or UniProtKB, gene IDs, groups of eukaryotic organisms, NCBI taxonomy IDs, the state of the proteins based on UniProtKB (Reviewed or Unreviewed), the "UniProt RTK status" and the classes of the RTKs (subfamilies and uncategorized RTKs). The search data can be combined as needed (with the options of: "AND/OR/NOT").
- Results - General: It is the main page where the results of a query for the user are presented. It contains the most basic information (accession number, gene id, gene names, protein names, species, subfamily and domains) about the RTKs and the user can select to download all or specific RTKs.
- Results - Download: The user can download different types of information for all the RTKs of his results or the selected RTKs.
- Results - RTK: This page presents all the information regarding a specific RTK which the user has selected. It also gives the ability of downloading all the information available for the selected RTK.
- Browse: The user can browse specific groups of RTKs of the TyReK database using a taxonomic group of organisms or a species or a specific class (subfamilies or uncategorized RTKs) or a domain or a GO term-term name.
- BLAST: The user can upload one or multiple protein sequences in FASTA format directly at the website or as a file and perform a protein BLAST (BLASTp) against the RTKs of the TyReK database (not including their isoforms).
- Manual: It contains detailed information about every page of the website of the TyReK database. It also provides examples for submitting queries to the database and interpreting their results, for downloading information from the database and for using the BLASTp utility.
- Download: The user can download all the information available by the TyReK database for all its RTKs or all sequences of the RTKs in the database including their isoforms or the sequences of the RTKs and their isoforms separately or the correspondence between the accession numbers of TyReK-DB and the accession numbers of UniprotKB.
- Contact: This page provides the e-mails of the people responsible for maintaining and updating the TyReK database and its website.

Supplementary References

30. The UniProt Consortium; Bateman, A.; Martin, M.-J.; Orchard, S.; Magrane, M.; Agivetova, R.; Ahmad, S.; Alpi, E.; Bowler-Barnett, E.H.; Britto, R.; et al. UniProt: The Universal Protein Knowledgebase in 2021. *Nucleic Acids Res.* **2021**, *49*, D480–D489, doi:10.1093/nar/gkaa1100.

33. Ashburner, M.; Ball, C.A.; Blake, J.A.; Botstein, D.; Butler, H.; Cherry, J.M.; Davis, A.P.; Dolinski, K.; Dwight, S.S.; Eppig, J.T.; et al. Gene Ontology: Tool for the Unification of Biology. *Nat. Genet.* **2000**, *25*, 25–29, doi:10.1038/75556.
34. The Gene Ontology Consortium; Carbon, S.; Douglass, E.; Good, B.M.; Unni, D.R.; Harris, N.L.; Mungall, C.J.; Basu, S.; Chisholm, R.L.; Dodson, R.J.; et al. The Gene Ontology Resource: Enriching a GOld Mine. *Nucleic Acids Res.* **2021**, *49*, D325–D334, doi:10.1093/nar/gkaa1113.
35. Huntley, R.P.; Sawford, T.; Mutowo-Meullenet, P.; Shypitsyna, A.; Bonilla, C.; Martin, M.J.; O'Donovan, C. The GOA Database: Gene Ontology Annotation Updates for 2015. *Nucleic Acids Res.* **2015**, *43*, D1057-1063, doi:10.1093/nar/gku1113.
36. O'Leary, N.A.; Wright, M.W.; Brister, J.R.; Ciufo, S.; Haddad, D.; McVeigh, R.; Rajput, B.; Robbertse, B.; Smith-White, B.; Ako-Adjei, D.; et al. Reference Sequence (RefSeq) Database at NCBI: Current Status, Taxonomic Expansion, and Functional Annotation. *Nucleic Acids Res.* **2016**, *44*, D733–D745, doi:10.1093/nar/gkv1189.
37. Schoch, C.L.; Ciufo, S.; Domrachev, M.; Hotton, C.L.; Kannan, S.; Khovanskaya, R.; Leipe, D.; Mcveigh, R.; O'Neill, K.; Robbertse, B.; et al. NCBI Taxonomy: A Comprehensive Update on Curation, Resources and Tools. *Database* **2020**, *2020*, baaa062, doi:10.1093/database/baaa062.
38. Bastian, F.B.; Roux, J.; Niknejad, A.; Comte, A.; Fonseca Costa, S.S.; de Farias, T.M.; Moretti, S.; Parmentier, G.; de Laval, V.R.; Rosikiewicz, M.; et al. The Bgee Suite: Integrated Curated Expression Atlas and Comparative Transcriptomics in Animals. *Nucleic Acids Res.* **2021**, *49*, D831–D847, doi:10.1093/nar/gkaa793.
41. Szklarczyk, D.; Gable, A.L.; Nastou, K.C.; Lyon, D.; Kirsch, R.; Pyysalo, S.; Doncheva, N.T.; Legeay, M.; Fang, T.; Bork, P.; et al. The STRING Database in 2021: Customizable Protein–Protein Networks, and Functional Characterization of User-Uploaded Gene/Measurement Sets. *Nucleic Acids Res.* **2021**, *49*, D605–D612, doi:10.1093/nar/gkaa1074.
42. Del Toro, N.; Shrivastava, A.; Ragueneau, E.; Meldal, B.; Combe, C.; Barrera, E.; Perfetto, L.; How, K.; Ratan, P.; Shirodkar, G.; et al. The IntAct Database: Efficient Access to Fine-Grained Molecular Interaction Data. *Nucleic Acids Res.* **2022**, *50*, D648–D653, doi:10.1093/nar/gkab1006.
43. Hermjakob, H.; Montecchi-Palazzi, L.; Lewington, C.; Mudali, S.; Kerrien, S.; Orchard, S.; Vingron, M.; Roechert, B.; Roepstorff, P.; Valencia, A.; et al. IntAct: An Open Source Molecular Interaction Database. *Nucleic Acids Res.* **2004**, *32*, D452-455, doi:10.1093/nar/gkh052.
44. Wang, Y.; Zhang, S.; Li, F.; Zhou, Y.; Zhang, Y.; Wang, Z.; Zhang, R.; Zhu, J.; Ren, Y.; Tan, Y.; et al. Therapeutic Target Database 2020: Enriched Resource for Facilitating Research and Early Development of Targeted Therapeutics. *Nucleic Acids Res.* **2019**, gkz981, doi:10.1093/nar/gkz981.
